# Distinguishing transient from persistent tactile agnosia after partial anterior circulation infarcts – Behavioral and neuroimaging evidence for white matter disconnection

**DOI:** 10.1016/j.nicl.2022.103193

**Published:** 2022-09-13

**Authors:** John H. Missimer, Eugenio Abela, Manuela Pastore-Wapp, Roland Wiest, Bruno J. Weder

**Affiliations:** a Laboratory of Biomolecular Research, Paul Scherrer Institute, Villigen, Switzerland; b Division of Neuroscience, Institute of Psychiatry, Psychology and Neuroscience, King's College London, London, United Kingdom; c Support Centre for Advanced Neuroimaging (SCAN), Institute for Diagnostic and Interventional Neuroradiology, Inselspital, Bern University Hospital, University of Bern, Bern, Switzerland

**Keywords:** Ischemic stroke, Tactile agnosia, Principal component analysis, MRI lesions maps, White matter disconnection

## Abstract

From a cohort of 36 patients presenting apperceptive tactile agnosia after first cortical ischemic stroke, 14 showed temporary impairment at admission. A previous multi-voxel analysis of the cortical lesions, using as explanatory variable the course of tactile object recognition performance over the recovery period of 9 months, partitioned the cohort into three subgroups. Of the 14 patients constituting two of the subgroups, 7 recovered from their impairment whereas 7 did not. These two subgroups could not be distinguished at admission. The primary aim of the present study is to present two assessments that can do so.

The first assessment comprises a pattern of behavioral measures, determined via principal component analysis, encoded in three tests: picking small objects, macrogeometrical discrimination and tactile object recognition. The receiver operating characteristic curve derived from permutation of the behavioral test scores yielded an 80% probability of correct identification of the patient subgroup and an 8% probability for false identification. As done with the permuted scores, the pattern could predict the persistence of affliction of new stroke patients with tactile agnosia.

The second predictive assessment extends our previous evaluation of cortical MRI lesion maps to include subcortical regions. Confirming our previous study, the lesions of the persistently impaired subgroup disrupted significantly the anterior arcuatus fasciculus and associated superior longitudinal fasciculus III in the ipsilesional hemisphere, impeding reciprocal information transfer between supramarginal gyrus and both the ventral premotor cortex and Brodmann area 44. Due to the importance of interhemispheric information transfer in tactile agnosia, we performed a supplementary analysis of tactile object recognition scores. It showed that haptic information transfer from the non-affected to the affected hands in the persistent cases partly restored function during the nine months, possibly following restoration of functional interhemispheric haptic information transfer at the border of posterior corpus callosum and splenium.

In conclusion, the combined findings of the cortical lesion at subarea PFt of the inferior parietal lobule and the associated subcortical tract lesions permit almost perfect prediction of persistent impairment of tactile object recognition. The study substantiates the need for combined analysis of both cortical lesions and white matter tract disconnections.

## Highlights

• Patterns of recovery from apperceptive tactile agnosia after first ischemic stroke.
• Behavioral pattern derived from PCA of three tests of tactile functionality.
• Neuroimaging shows disruption of white matter tracts in ipsilesional hemisphere.
• Tracts include anterior arcuatus fasciculus and superior longitudinal fasciculus III.
• Haptic information transfer between hemispheres supports recovery.

## 1 Introduction

An important consequence of the residual hemiparesis suffered by up to 50 percent of stroke survivors older than 65 years of age is limitation of activity, e.g. disturbed daily sensori-motor hand skills. These complex skills should be distinguished explicitly from elementary body functions (Miller et al. 2010). Partial dexterity and perceived participation after moderate and mild stroke have been shown by Ekstrand and colleagues to be specifically important resources for rehabilitation of the upper extremity (Ekstrand et al., 2016). In particular, manual dexterity involves motor control during active touch, such as the grasping of objects, in which finger movements are both partly independent and temporally synchronized (Ekstrand et al., 2016, Térémetz et al., 2015). Specific tasks required to discriminate macroscopic somatosensory stimuli seem to be very vulnerable, causing particular activity limitations (Carey and Matyas, 2011, Kim and Choi-Kwon, 1996, Han et al., 2002, Smith et al., 1983, Tyson et al., 2008, Welmer et al., 2008). In the case of object shape recognition, the limitations are due to mechanisms underlying the dynamic exploratory finger movements (Krammer et al. 2020). The disruption of tactile object recognition (TOR) can be due to either apperceptive or associative disorders, and affects the recognition of macroscopic and microscopic object features or qualities like hardness or softness amongst others. Investigations of these disorders have been limited until now to reports of single cases or small cohorts (Bohlhalter et al., 2002, Caselli, 1991, Hömke et al., 2009, Kitada et al., 2019, Nakamura et al., 1998, Platz, 1996, Reed et al., 1996, Saetti et al., 1999, Schendel et al., 2021, Veronelli et al., 2014).

Moreover, marked variability in long-term sensori-motor outcomes hinders accurate individual predictions, particularly in severely affected individuals (Stinear, 2010, Stinear et al., 2017). Differences in brain structures and associated functions, as assessed by lesional and functional neuroimaging studies, might provide insight into heterogeneity in recovery and lead to refined predictive models (Marshall et al., 2009, Rehme et al., 2015, Stinear et al., 2017).

In a prospective and longitudinal study over 9 months of 36 patients with stroke of the sensori-motor cortices (Abela et al., 2019), we evaluated behavioral measures and derived lesion maps from T1-weighted (T1w) MRIs. The seven measures included age, NIH score, the subset of the Jebsen-Taylor Hand Function test denoted picking small objects (PSO), cutaneous pressure perception threshold (PPT), micro- and macrogeometrical discrimination (MIC and MAC), and tactile object recognition (TOR).

The performance of 22 patients, denoted TN, i.e. TOR normal, did not deviate significantly from healthy controls whereas the remaining 14 patients presented marked apperceptive tactile agnosia, exhibiting consistently disrupted haptic object recognition during dynamic finger exploration caused by distinct anteroparietal lesions (Binkofski et al. 2001). After nine months, 7 of these 14 patients, the recovered subgroup, performed the task satisfactorily whereas the remaining 7 patients, the persistently impaired subgroup, could not. The recovered subgroup will be hence denoted RTI, i.e. recovered from TOR impairment, and the persistently impaired subgroup PTI, i.e. persistent TOR impairment.

We observed in the study of Abela et al. (Abela et al., 2019) that a multi-voxel pattern analysis of the cortical lesion pattern, using as target variable the course of TOR performance over the recovery period of 9 months, could replicate with high significance the patient TOR performance trajectories and distinguish between TN and PTI subgroups. A *meta*-analysis of tactile activation in healthy subjects revealed six areas involved in the lesion network common to the entire patient cohort. These areas included a subarea of the inferior posterior frontal cortex (Clos, Amunts, Laird, Fox, and Eickhoff 2013), Area 4a of the precentral gyrus, Area 1 of the postcentral gyrus, area hIP2 of the intraparietal sulcus, area PFt of the supramarginal gyrus and Area OP1 (SII) of the parietal operculum. Specific to the PTI subgroup was involvement of the tactile network containing PFt, suggesting disruption by the ischemic lesion of the motor mirror network between area PFt and the target area of inferior posterior frontal cortex. Two mechanisms of permanent impairment may be inferred: (1) a direct ischemic tract lesion within the white matter and (2) a secondary axonal tract lesion due to neuronal loss in the seed structure of PFt subarea. These considerations suggested our working hypothesis that subcortical lesions affecting the anterior arcuate fascicle and superior longitudinal fascicle III might be decisive components of the permanent impairment in the PTI subgroup.

The two subgroups, RTI and PTI, could not be distinguished at admission. As our primary aim, we intended to demonstrate on the basis of the behavioral measures and MRI lesion maps of the two subgroups a means of predicting at admission which patients will remain impaired: (1) Reassessment of the behavioral measures via principal component analysis, PCA, yielded a pattern encoded in PSO, MAC and TOR performance scores that distinguished the two patient subgroups. To test the statistical reliability of the pattern, we derived a test set by permutation of the scores consistent with the respective degree of impairment. (2) Reevaluation of the lesion maps consisted of determining subcortical regions that, in addition to the cortical sites explored in our previous study, distinguish the PTI and RTI patient subgroups. In light of the cortical pattern, we expected disruption of tracts connecting supramarginal gyrus with ventral premotor cortex, vPMC, and Brodmann area 44, BA 44, mainly in the PTI subgroup.

Since *trans*-hemispherical tactile information transfer is known to be dysfunctional after ischemic stroke (Bohlhalter et al., 2002, Hömke et al., 2009, Schendel et al., 2021), we also performed as secondary aim an exploratory analysis of TOR scores in haptic information transfer from the non-affected to the affected hands and vice versa in all patient subgroups during the nine months. This analysis of matching, a typical feature of haptic information processing (Reed et al., 1996), could contribute to the understanding of prevailing physiological conditions within the two subgroups and be of significance for rehabilitation. Specifically, we investigated behavioral interaction between hemispheres and associated affection of corpus callosum.

## 2 Participants and methods

### 2.1 Participants

As described in our previous study (Abela et al., 2019), we recruited stroke patients from two comprehensive stroke centres in Switzerland (Department of Neurology, Kantonsspital St. Gallen, and University Department of Neurology, Inselspital, Bern). Inclusion criteria were: (1) first ever ischemic stroke, (2) clinically significant hand plegia or paresis as leading symptom, and (3) involvement of the pre- and/or postcentral gyri confirmed on diffusion-weighted (DWI) and fluid attenuated inversion recovery (FLAIR) MRI. Additional involvement of frontal, parietal and opercular regions was accepted but not selected for. Exclusion criteria were: (1) aphasia or cognitive deficits severe enough to preclude understanding the study purposes or task instructions, (2) prior cerebrovascular events, (3) occlusion of the carotid arteries on MR–angiography, (4) purely subcortical stroke b/c not directly related to cortical functions, and (5) other medical or neurological conditions interfering with task performance. According to the Edinburgh Handedness Questionnaire, 33 patients were right-handed with laterality quotients (LQ = (R-L)/(R + L) * 100) ranging between 43 and 100 and median 89; three were ambidextrous with laterality quotients ranging between 30 and 40 (Oldfield, 1971). The study received ethical approval from the institutional review boards of both research sites. All participants gave written informed consent before enrolment, according to the Declaration of Helsinki (World Medical Association, 2013). Of the cohort of 36 patients, 22 performed as well as the healthy controls on a test of tactile object recognition (TOR) within three months after admission. The 14 remaining patients, of which 7 did not recover significantly even after nine months, are the main subject of this study. For detailed demographic and clinical characteristics see (Abela et al., 2019).

### 2.2 Methods

The longitudinal study of the entire cohort consisted of three main visits: an examination at admission within the first two weeks after stroke and follow-up examinations at 3 and 9 months. We additionally performed monthly control visits to monitor recovery of hand function, including a specific sensori-motor task according to Jebsen et al. (1969). For the present analysis, we used behavioral and neuroimaging data acquired at admission.

#### 2.2.1 Predictive assessment of behavioral data

The assessment of behavioral measures seeks a pattern of measures, determined via PCA, which distinguishes between the RTI and PTI subgroups.

The behavioral data submitted to PCA included age, NIH score, the subset of the Jebsen-Taylor Hand Function test denoted picking small objects (PSO), cutaneous pressure perception threshold (PPT), micro- and macrogeometrical discrimination (MIC and MAC), and tactile object recognition (TOR).

As described in (Abela et al., 2019), we assessed stroke severity using the NIH Stroke Scale (Brott et al., 1989). Sensori-motor functions of both hands were assessed. Fine motor skill was evaluated using the Jebsen-Taylor Hand Function test (Jebsen et al., 1969): On the basis of previous analyses showing the picking small objects (PSO) subtest to be the most informative with respect to motor skill recovery (Abela et al., 2012), we focused on this test. PSO requires the patient to pick six small objects consisting of 2 paper clips, 2 bottle caps and 2 coins with one hand, and drop them as fast as possible into an empty can in front of them. Performance is measured in seconds. We quantified cutaneous pressure perception threshold (PPT) with graded monofilaments as described in (Dyck et al., 1993) and measured micro- and macrogeometrical discrimination (MIC and MAC, respectively), as previously reported (Bohlhalter et al., 2002, Hömke et al., 2009, Weder et al., 1998). MIC was determined by requiring the blindfolded subject to choose which of two grated plastic surfaces differing in roughness was rougher; synthetic surfaces with different grating profiles of alternating grooves and ridges having spatial periods of 1.0 mm and 1.1 mm were presented sequentially. For MAC, we required the subject to choose which of two aluminium parallelepipeds of identical volumes (11.5 cm^3^) but differing oblongness was longer; pairs of parallelepipeds with a difference of 3.97 mm in the long axes were presented sequentially. For both tasks, we presented 24 counterbalanced pairs of objects and quantified performance as the proportion of correct decisions. The differences in roughness and oblongness were fixed at a threshold that yielded 90% correct decisions in healthy controls (Weder et al., 1998, Hömke et al., 2009). Healthy volunteers are able to discriminate differing oblongness with a probability of 0.95 and 95% confidence level interval between 0.92 and 0.97, and differing roughness via scanning movements with a probability of 0.9 and 95% confidence level interval between of 0.88 – 0.92.

To evaluate TOR performance, we presented participants with 30 common everyday objects which they explored with one hand while blindfolded (Bohlhalter et al., 2002). Objects were empirically chosen from household items that could be easily grasped, held and explored with one hand. The objects used are listed in Supplementary Material. We sequentially presented each object to the patient’s hand, allowing a maximum of 10 s for manual exploration and object recognition and 5 s pause after each presentation. One run was performed per hand. Object presentations and order of hands were pseudorandomized over subjects and visits. The TOR performance data are summarized in Table 1. The objects are identified in Table S1.

**Table 1:** Tactile object recognition (TOR) after first ischemic stroke. Shown are the performance scores for tactile object recognition: the number of correctly identified out of 30 household objects within 10 s with affected hand (aH) and unaffected hand (uH). The results are displayed for admission and after 3 and 9 months. The first entry is the median of correct identification for the group; in parentheses is the range. Statistical comparison of two sequences were performed with the Mann-Whitney 2-tailed test and all sequences with the Kruskal-Wallis H test. Only p-values surviving after correction for multiple comparisons are indicated.

		UH	AH	AH vs UH
Group	time	Median (range)	Median (range)	M−W 2-tail
			TOR 0	
TN (n = 22)	admission	30 (28–30)	30 (24–30)	n.s.
RTI (n = 7)		30 (6–30)	4 (0–20)	z = 2.7, p < 0.011
PTI (n = 7)		26 (10–30)	0 (0–3)	z = 3.1, p < 0.002
Kruskal-Wallis		n.s.	H = 26.6, p < 0.0001	
			TOR 3	
TN	3 months	30 (28–30)	30 (28–30)	n.s.
RTI		28 (22–30)	15 (18–28)	n.s.
PTI		29 (22–30)	4 (0–6)	z = 3.1, p < 0.002
Kruskal-Wallis		H = 7.3, p < 0.05	H = 26.4, p < 0.0001	
			TOR 9	
TN	9 months	30 (30–30)	30 (27–30)	n.s.
RTI		29 (28–30)	25 (19–30)	n.s.
PTI		30 (23–30)	3 (0–10)	z = 3.1, p < 0.002
Kruskal-Wallis		n.s.	H = 23.7, p < 0.0001	

In (Abela et al., 2019), we classified patients into subgroups based on the number of correctly identified objects at the end of the study. The TN subgroup included patients who attained TOR performance at admission within the limits of healthy controls, i.e., 26–30 objects. The RTI subgroup included patients who achieved after nine months at least 50% correct recognition (n > 15) and the PTI subgroup consisted of patients who achieved <50% (n < 15). At admission, the ranges of performance scores for the two subgroups overlapped. One patient classified in Abela et al. (2019) as TN according to its nine-month recovery trajectory was reclassified as RTI in this analysis due his TOR score at admission. Distinguishing the seven patients of the RTI subgroup from the seven of the PTI subgroup using data acquired at admission is the object of this investigation. The clinical and behavioral data for the three patient subgroups are summarized in Table 2.

**Table 2:** Clinical and behavioral data for the three patient groups. The distribution comprises total number of patients in the group, followed by the number of males and females. Age, lesion volume and the six behavioral scores were used in the principal component analysis. In these, the suffix 0 denotes score at admission. The entries in the first row are the medians, in the second row are the ranges.

group	distribution	age [years]	lesvol [cc]	NIHSS score	PPT 0 g/mm^2^	MIC 0 number	MAC 0 number	PSO 0 [sec]	TOR 0 number
TN	22/18/4	66.5 41–80	4.5 0.5–61	4 1–7	10.0 7.0–59.2	20.5 10–24	23 19–24	10.6 5.3–26.9	30 24–39
RTI	7/5/2	75 51–82	18.5 3.4–44.1	4 3–6	59.2 9.0–178	10.0 0–22	14 0–20	22 10.9–76.1	4 0–20
PTI	7/6/1	54 49–70	44.1 22–272.3	6 3–14	158.2 37.3–177	0 0–13	0 0–15	33.1 12.3–67.1	0 0–3

As predictors of TOR impairment nine months after stroke, eight measures derived from behavioral data acquired at admission of the fourteen patients were submitted to a sequence of PCAs constituting an Occam’s rasor. As summarized in Table 1, the initial measures included age, lesion volume, NIH, MIC, MAC, PPT, PSO and TOR. Before PCA, the data, with the exception of age, lesion volume and NIH were converted to z-scores using gender and hand-matched data from the healthy control group described in Abela et al. (2012), such that more negative scores indicated increased impairment. Z-scores for age and lesion volume were computed from the patient cohort; the original NIH scores were used since they are comparable to the z-scores. Three of the fourteen patients exhibited plegic fingers at admission and were unable to explore the objects by active touch as required by PSO. They could complete the task after one month; the intercepts of their recovery trajectories determined in Abela et al., (2012) were used to model times for PSO at admission in these patients. These are conservative estimates compared to the longest times measured for PSO in the remaining patients or any longer (infinite) time. With the MATLAB function *pca.m*, PCAs of the behavioral measures were computed. Each PCA yielded a number of components equal to the number of measures analyzed, percent of variance explained by a component, expression coefficients describing the degree to which a behavioral measure contributes to the component, and 14 patient scores describing the degree to which each patient contributes to the component. The expression coefficients provide an orthonormal basis for the measures, implying that patient scores of other patients can be computed by projection. The principal components were ordered according to the percentage of variance explained; components contributing to a cumulative percentage of 80%, usually the first two, were admitted to further analysis. To be considered salient, the principal component must have exhibited significant correlation, p < 0.05, between the patient PC scores and the TOR performance scores measured at nine months. The Matlab function *kruskal*–*wallis.m*, a nonparametric one-way analysis of variance appropriate for small sample sizes, evaluated the significance of discrimination between RTI and PTI patient PC scores. Further analysis of the patient score distribution estimated the discrimination threshold.

The application of Occam’s razor utilized the component expression coefficients. Behavioral measures contributing dominant expression coefficients were retained in the succeeding analysis. As presented in Results, the PCA of the original eight measures showed the dominance of MIC, MAC, PSO, and TOR. The PCA of those four measures showed a less clear pattern, indicating two further PCAs of MIC, PSO and TOR and MAC, PSO and TOR, respectively. The last PCA will be the focus of Results.

In order to generate data to validate the most discriminating PCA, we performed permutation testing. The validation consisted of refining the estimates of the discrimination threshold and the rates of false positives and negatives. The set of behavioral measures: MAC, PSO and TOR, were permuted separately for each RTI and PTI subgroup, yielding 7^3^ = 343 combinations of measures for each subgroup. To avoid extreme combinations, only those were included in further analysis that yielded Mahalanobis distances - computed using the MATLAB function *mahal.m* as the Euclidean distances from the mean of the TOR normal subgroup in the three-dimensional space of MAC, PSO and TOR performance scores - lying within the range of distances determined by the original set of measures. From each accepted permutation was computed a simulated patient score by projection of the expression coefficients. By applying the MATLAB function *perfcurve.m*, the combined distributions of simulated patient scores generated a receiver operating characteristic (ROC) curve that determined the optimal operating point, ie threshold, for discrimination and the rates of true and false positives. These could then be compared with the original patient score distribution.

#### 2.2.2 Predictive assessment of neuroimaging data

The assessment of neuroimaging data seeks a neuroanatomical lesion pattern, which distinguishes between the RTI and PTI subgroups. The disruption of white matter tracts implied by the cortical lesions plays an essential role in the pattern.

Acquisitions at admission were carried out of the 36 patients in the first 9 cases with 1.5 Tesla on a clinical whole-body MR scanner (SIEMENS Magnetom Vision) using the standard head coil. MRI studies were performed in the remaining cases with 3 Tesla on a clinical whole body MR scanner (SIEMENS Trio). All follow-up scans were carried out with 3 Tesla on the latter scanner as described in (Abela et al., 2019). The T1-weighted, T1w, and diffusion-weighted images, DWI, of both centers were processed as described below. The DWI data of one patient belonging to the TN subgroup were corrupted and could not be recovered. This patient was excluded from the lesion analyses, reducing the number patients in the neuroimaging analysis to 35.

As described (Abela et al., 2019), cortical lesions were manually traced by one author (EA) on DWI scans using MRIcron (https://www.nitrc.org/projects/mricron/), yielding binary lesion masks in individual anatomical space. To avoid bias, lesion tracing was performed without knowledge of the results of the behavioral data analysis. DWI images and binary lesion masks were co-registered to the T1w images using SPM12 (https://www.fil.ion.ucl.ac.uk/spm/software/spm12/) for MATLAB (R2016b, The MathWorks, Inc., Natick,Massachusetts, United States). We then segmented and normalized the T1w images into Montreal Neurological Institute (MNI) space by means of unified segmentation-normalisation (Ashburner & Friston, 2005). Using the Clinical Toolbox for SPM12 (Rorden et al., 2012), cost-function masking was applied to exclude damaged areas from the calculation of the normalisation parameters (Andersen et al., 2010, Brett et al., 2001). These parameters permitted the lesion masks to be warped into MNI space. Lesion masks were finally smoothed with an isotropic 4 mm^3^ Gaussian kernel to compensate for interpolation errors (Abela et al., 2012). Cortical lesion overlap masks were generated using MRIcron. Neuroanatomical localisation was done with the SPM Anatomy Toolbox (Version 2c.c, http://www.fil.ion.ucl.ac.uk/spm/ext/#Anatomy). This toolbox relies on the probabilistic cytoarchitectonical Jülich atlas of Eickhoff et al., 2005.

##### 2.2.2.1 Tractography

Following registration to MNI152 space, disconnectome maps of all 35 patients were calculated from the cortical lesion maps using the BCBtoolkit (Foulon et al. 2018; http://www.toolkit.bcblab.com). Based on the diffusion weighted imaging data sets for 10 healthy controls (Rojkova et al., 2016), the tractography for each lesion could be estimated and transformed to visitation maps (Thiebaut de Schotten et al., 2011). An overlap map derived from the normalized visitation map of each healthy subject resulted in the disconnectome map, in which each voxel accounts for the inter-individual variability of the controls yielding a probability of disconnection (Thiebaut De Schotten et al., 2015). Of the 35 patients, the majority: 11 TN and 5 each of PTI and RTI subgroups, presented cortical lesions in the right hemisphere. The disconnectome maps of these patients were submitted to statistical analysis using the threshold-free cluster enhancement toolbox, TFCE (http://www.neuro.uni-jena.de/tfce/) of SPM-12. Contrasts between PTI and TN subgroups and between RTI and TN subgroups were computed using the permutation-based non-parametric T-test and the requirement pFDR < 0.05 corrected across contrasts (Nichols and Holmes, 2001).

Using Tractotron software included in the BCBtoolkit, we also determined for all 35 patients the severity of the disconnection of the tract damaged by the lesion (Thiebaut De Schotten et al., 2015). For statistical analysis, the tracts of the 21 patients presenting lesions in the right hemisphere and those of the 14 patients with lesions of the left hemisphere were classified together as affected hemisphere, AH. For a given lesion, Tractotron provides a probability and proportion of disconnection for almost all known tracts (Foulon et al., 2018). The probability relates the voxels of a lesion to the atlas of white matter tracts defined by the healthy controls. The voxels of the lesion with the highest degree of overlap define the probability of disconnection. It is assigned a value of one if a voxel of the lesion is common to all white matter tracts in the atlas. By assumption, a probability >0.5 indicates disconnection. The proportion expresses the number of damaged voxels in the tract divided by the total volume of the tract. We regarded it as the measure more clearly reflecting the extent of damage to the entire tract, and performed one- way ANOVAs and t-tests of the proportions to evaluate the differences between patient groups.

##### 2.2.2.2 Cortical lesions

In (Abela et al., 2019) we calculated the tactile network performing a *meta*-analysis as related to functional MRI data using the Neurosynth database (http://www.neurosynth.org) (Yarkoni et al., 2011). Submitting the search term “tactile” and excluding all studies that did not use 3 T MRI, did not investigate somatosensory processes, or reported results of patient populations yielded 45 studies. Synthesis of these studies yielded a bi-hemispheric map of activations uniquely associated with the term “tactile” (Abela et al., 2019). The significance threshold of this map was z = 3.89, corresponding to a false-discovery rate (FDR) of q < 0.01. The results are available at https://osf.io/n97cb/. Our motivation for using Neurosynth was solely to derive regions-of-interest (ROI) that would meaningfully constrain the voxel-space used for multi-voxel pattern analysis (MVPA) - in essence, to design a spatial filter driven by *a priori* knowledge of fMRI activations associated with tactile object recognition (TOR). Selecting only studies that reported results from *healthy* populations, we found a *normative* set of ROIs that avoided the biases inherent in a diseased population such as alterations in the BOLD response, alternative cognitive strategies during task performance, and other mechanisms such as plasticity or diaschisis. Our pragmatic goal was the delineation of regions specific to TOR by excluding functionally irrelevant areas of the complete lesion map. Applying permutation tests and Leave One Subject Out cross-validation, we found that MVPA achieved a highly significant reproduction of the individual TOR performance recovery scores (Abela et al., 2019).

In analogy with Abela et al. (2919), we performed using this map a standard univariate voxel-behavior analysis of the lesioned areas of the RTI and PTI subgroups, comparing them with the TN subgroup, by means of the Liebermeister measures (Bates et al. 2003, Rorden et al., 2007) as implemented in NiiStat (https://www.nitrc.org/projects/ niistat).

#### 2.2.3 Analysis of tactile matching tasks

We also performed as secondary aim an exploratory analysis of TOR scores in haptic information transfer from the non-affected to the affected hands and vice versa in all patient subgroups during the nine months.

To investigate tactile information transfer, two types of matching tasks were performed additionally in RTI and PTI patient groups: tactile-tactile matching with the affected (i) and non-affected hand (ii), tactile matching of objects involving the non-affected hand after presentation of objects to the affected hand (iii), and reverse (iv). In each of these tasks, the replica for 10 objects had to be identified from among five different objects. A period of 10 s was allowed for tactile exploration and object identification. The matching tasks were performed also at admission, three and nine months. Mann Whitney and Friedman statistical tests were performed to assess differences between non-affected and affected hands for each group and changes of each hand with time.

## 3 Results

### 3.1 Predictive assessment of behavioral data: Principal component analysis

**Fig. 1:**
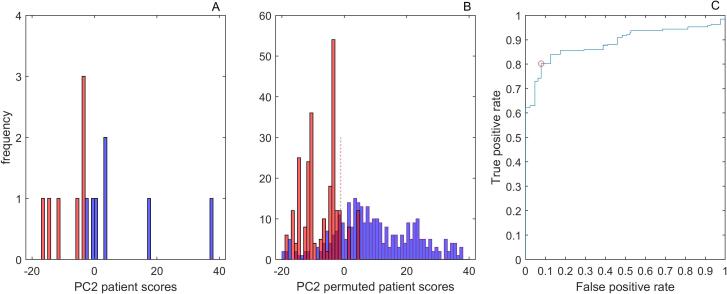
Distributions of patient scores for PC2 of the PCA of MAC, PSO and TOR for the original patient scores (left panel) and the permuted behavioral scores (mid panel). Red denotes the PTI subgroup and blue the RTI subgroup. The right panel shows the receiver operating characteristic curve (ROC) for the PC patient scores derived by permutation. (For interpretation of the references to color in this figure legend, the reader is referred to the web version of this article.)

Applying Occam’s razor, an hierarchy of principal component analyses determined a pattern of PSO, MAC and TOR expression coefficients that distinguished between RTI and PTI patient subgroups as determined by the correlation of patient scores with TOR task performance at nine months. Including the eight behavioral measures of Table 2: age, lesion volume, NIH score, MAC, MIC, PPT, PSO and TOR, at admission, the first PCA found a component with significant correlation in which the expression coefficients for MAC, MIC, PSO, and TOR were clearly dominant and the patient score distributions showed marked overlap. The PCA of the dominant four measures yielded a second component significantly correlated with TOR 9, but again marked overlap of the PTI and RTI subgroup patient scores. This PCA exhibited a dominance of MIC, PSO and TOR and the Kruskal-Wallis analysis showed improved discrimination, but overlap of the distributions was also evident. The PCA of these three dominant measures yielded improved statistical discrimination between the two patient groups, with χ^2^ = 9.0 corresponding to p < 0.01, but markedly overlapping subgroup patient scores. Moreover, the multiple linear regression of TOR recovery trajectories versus seven of the eight behavioral measures, excluding only PPT, of Abela et al. (2019) showed that MAC and PSO were the significant measures explaining almost 90% of the variance. We thus performed a PCA including only MAC 0, PSO 0 and TOR 0. The second principal component explained 30% of the variance and yielded a significant correlation, p < 0.008, with TOR performance nine months after admission. As indicated in Fig. 1, the overlap of patient scores was minimal. The Kruskall-Wallis nonparametric test indicated significant discrimination between the two patient groups, with χ^2^ = 9.8 and p < 0.002; a threshold of −3 yielded perfect discrimination. Table 3 summarizes the hierarchy of analyses and Supplementary Material presents additional details concerning the PCA. As Table S5 shows, the dominant expression coefficient of the 1st PC is provided by PSO, which is associated with lateralized fine motor skill, whereas TOR, associated with shape and texture recognition, yielded the dominant expression coefficients of the 2nd PC. This is also true for the PCA comprising PSO, MIC and TOR (not shown).

**Table 3:** Summary of PCA analyses. The first column identifies the PCA, the second the salient component correlating significantly with TOR 9, the following eight columns show the expression coefficients computed in the PCA, the last two columns summarize the Kruskal-Wallis one-way ANOVA distinguishing RTI from PTI.

	PC	age	Les vol	NIH	pso	mac	mic	ppt	tor	KW χ^2^	KW p
PC8	3	0.054	−0.117	−0.149	−0.473	0.384	0.406	−0.213	0.616	3.92	0.048
PC4	2				0.695	−0.195	−0.423		−0.547	6.86	0.009
PC3 mic	2				−0.434		0.570		0.698	9.02	0.003
PC3 mac	2				−0.505	0.383			0.774	9.8	0.002

**Fig. 2:**
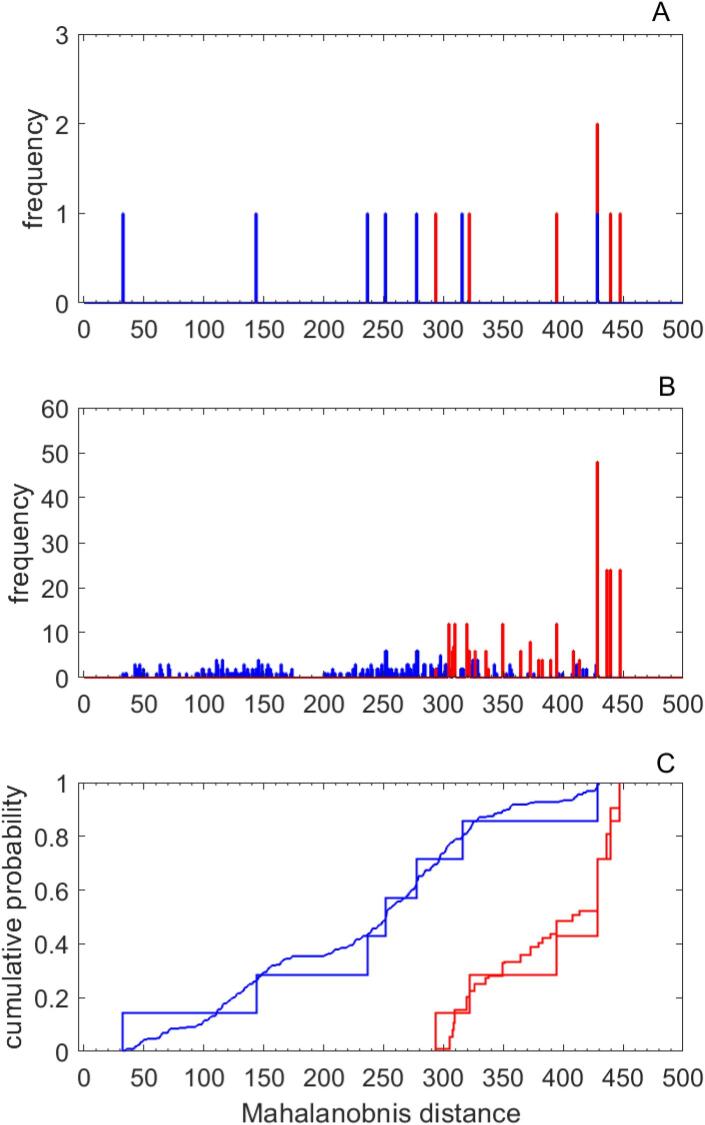
In the upper panel are plotted the distributions of Mahalanobis distances, i.e. the distances from the mean of the TN subgroup in the three-dimensional space of MAC, PSO and TOR scores, for the PC patient scores of the RTI and PTI subgroups. In the middle panel are the analogous distributions for the PC patient scores derived by permutation. In the lower panel are displayed the cumulative probability of the distance distributions for the original and derived scores for the two subgroups. Red denotes the PTI subgroup and blue the RTI subgroup. (For interpretation of the references to color in this figure legend, the reader is referred to the web version of this article.)

Regarding the test measures generated by permutation, we required that the permuted sets of three measures yield Mahalanobis distances that fall within the limits of the original sets. These limits were [32 429] for the RTI group and [293 448] for the PTI group, implying 319 and 252 sets of the total of 343, respectively. The mid graph of Fig. 2 shows the distribution of Mahalanobis distances computed for the test data; these are the distances from the mean of the TOR normal subgroup in the three-dimensional space of MAC, PSO and TOR measures. The lower panel shows the cumulative probability with respect to distance and comparison with the original patient scores. The cumulative probabilities showed good agreement, indicating that the permuted test measures yielded a realistic representation of Mahalanobis distances. The mid panel of Fig. 1 shows the distribution of patient scores derived from the permuted performance scores; the distribution reflects well the distribution of original behavioral scores shown on the left panel of Fig. 1 but displays a realistic overlap of RTI and PTI patient scores. The right panel of Fig. 1 shows the receiver operating characteristic curve (ROC) for the computed patient scores. The optimal operating point implies that the optimal threshold for distinguishing RTI from PTI subgroups – shown by dotted line in the panel - is achieved with the PC patient score, −1.09, yielding a true positive rate of 0.803 and a false positive rate of 0.079, implying a Youden Index, J = 0.723. These rates imply 256 of 319 true occurrences and 20 of 252 false occurrences in the range above the threshold; the balanced accuracy for discrimination is 0.9. For the original data of seven patients in each category, this optimal threshold yields 1 false positive and 6 true positives, corresponding to a false positive rate of 0.125 and a true positive rate of 1. Including all the permuted patient scores without restriction reproduces the optimal threshold, but yields a true positive rate of 0.767 and a false positive rate of 0.169.

### 3.2 Predictive assessment of neuroimaging data: Lesion analysis and tractography

A neuroanatomical pattern, consisting of a cortical lesion at subarea PFt and significant disruption of the subcortical anterior arcuate fasciculus and associated superior longitudinal fasciculus III in the ipsilesional hemisphere permits almost perfect prediction of persistent tactile impairment and distinguishes the two subgroups at admission.

Because the MRI data were acquired at different magnetic field strengths (see Methods), we controlled for a confounding influence of scanner type on the results. We visually confirmed that lesion maps of patients scanned at 1.5 T overlapped the chronic infarct cores seen on follow-up 3 T MRIs. We determined that the lesion volumes were not systematically different between the 9 patients scanned with 1.5 T and the 26 with 3 T: U = 89.5 yielding p < 0.25. Finally, a multi-voxel pattern analysis failed to identify scanner type from lesion maps alone: AUC = 0.5, balanced accuracy 53.4%, p < 0.29 (Abela et al., 2019).

**Fig. 3:**
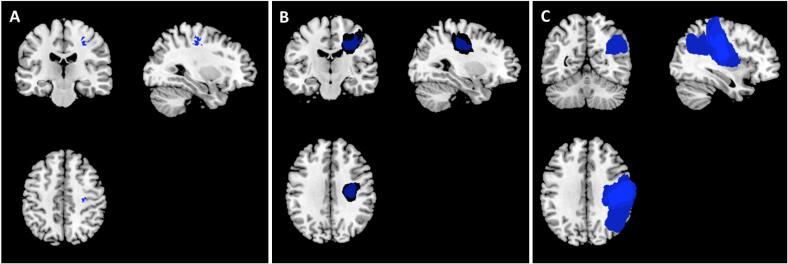
Lesion conjunction maps. Maps show voxels for which lesion maps of at least 71.4% of patients overlap: 15 of 21 for the N subgroup in the left panel (A), 5 of 7 for the RTI subgroup in the mid panel (B) and 7 of 7 for the PTI subgroup in the right panel (C). Images are in neurological convention: left side of the image corresponds to left side of the brain. The lesion core (25/35 patients) was located in the depth of central sulcus; in the N and RTI subgroups the overlap is not maximal due to the greater spatial variance of smaller lesions.

In Fig. 3 are shown representative planes of lesion conjunction maps reconstructed from baseline diffusion weighted imaging for all three patient groups. According to the probabilistic cytoarchitectonical Jülich atlas (Eickhoff et al., 2005), the lesion core for the TN subgroup was located in the motor Area 4p. The lesion cores of the PTI and the RTI group had in common the motor Area 4p, PSC2 and PSC3a/b; the core of the PTI group also included IPL PF and PFcm as well as OP1, PSC1. Of these, OP1 and IPL PF are essential elements of the tactile network.

Table S9 displays complete results of white matter tract lesions using the BCBtoolkit. Affecting only the ipsilesional hemisphere, 25 tracts of the PTI subgroup and 22 tracts of the RTI subgroup contain voxels surpassing the critical probability threshold of P > 0.5. The proportion of overlap with white matter tracts in PTI and RTI subgroups yielded a significant higher lesion load in the former (2-tailed *t*-test, p < 0.01). The anterior arcuate fasciculus, superior longitudinal fasciculus (SLF III) and corpus callosum of the PTI subgroup, a focus of our study, exhibited maximal voxel probablilities, P = 1. The contralesional hemisphere showed no indication of disconnection: P = 0. This contrast confirms the association of the specifically affected white matter tracts with the stroke and belies the premise of unspecific white matter lesions.

**Fig. 4:**
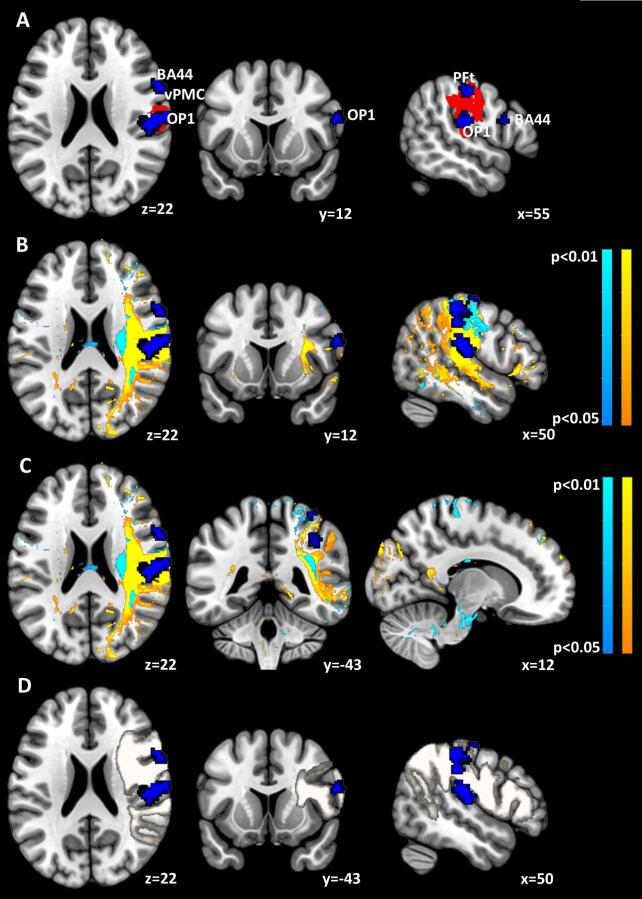
Synopsis of cortical and white matter lesions for PTI and RTI subgroups**.** In the panel A is shown in red the cortical lesion of the PTI subgroup determined to be significant compared to the TN subgroup using the Liebermeister test for binary data. The blue areas depict constituents of the *meta*-analytic tactile network disrupted by the lesion; subareas PFt and OP1 as well as the inferior posterior BA44 at the junction to the ventral PMC. These latter areas are also shown in panels B, C and D. In panel B are delineated the disconnectome maps derived from contrasts of the PTI with the N subgroups in warm colors and of the RTI with the N subgroups in cool colors. The threshold level, pFDR < 0.05, is corrected across contrasts. In panel C a lesion of the crossing fibre tracts in the right paracallosal area, entering corpus callosum (coronal slice) is discernible in the PTI subgroup at the border between the posterior corpus callosum and splenium. In panel D are shown the constituents of the damaged *meta*-analytic network juxtaposed with the anterior arcuate fasciculus, depicted as a circumscribed white ROI, as represented by the BBC Toolkit. (For interpretation of the references to color in this figure legend, the reader is referred to the web version of this article.)

Fig. 4 elaborates the effects of the lesions on associated neuronal networks and white matter tracts. In the representative planes of the upper panels, the red denotes voxels determined by the Liebermeister comparison of the PTI with the TN subgroups to exceed a significance threshold, z > 3.98, corresponding to p < 0.05 per permutation testing. The color blue represents three clusters of the *meta*-analytic map of 45 functional magnetic resonance imaging (fMRI) studies associated with the term “tactile” and intersecting the lesion maps in red. This *meta*-analysis revealed in total eight clusters that attained significant voxel-wise z-scores exceeding 3.89, corresponding to a false-discovery rate, q < 0.01, per permutation testing. As discussed in Introduction and Methods, six of these clusters contributed essentially to prediction of TOR performance over the course of the study (Abela et al, 2019). They comprised the inferior posterior Broca subarea of BA44 described by (Clos et al., 2013) with center-of gravity MNI coordinates x = 59,y = 11,z = 23; Area 4a of the primary motor cortex with coordinates (34,-31,71); Area 1 of the primary sensory cortex (36,-43,64); Area 1 of the primary sensory cortex and PFt of the inferior parietal lobule (52,-24,57); area hIP2 of the intraparietal area (40,-41,44); and area OP1 (SII) of the parietal operculum (55,19,19). The two areas not contributing to this lesional neural network were regions of the dorsal premotor and insular cortex.

The middle panels show composite disconnectome maps representing contrasts between the PTI and TN subgroups and between the RTI and TN subgroups. Computed using the BCB Toolkit for each of the patients, the 35 individual disconnectome maps were submitted to univariate SPM analysis of the contrasts between the 5 patients of the PTI or RTI subgroups and the 11 patients of the TN subgroup, respectively. A permutation-based analysis with threshold-free cluster enhancement (Nichols and Holmes, 2001) with significance threshold p < 0.05 and pFDR < 0.025 corrected across contrasts yields the areas shown for the PTI and RTI contrasts. Note that the more extensive white matter tract disconnection in the PTI contrast affects severely the gap between the PFt subarea and the vPMC/BA44. In addition, the coronal slice indicates a disconnection of the crossing fibre tracts within the paracallosal zone and corpus callosum that is mainly discernible in the PTI contrast. This disconnection lays at the border of the posterior corpus callosum and splenium, a crucial region for haptic information transfer (Fabri and Polonara, 2013).

Finally, the lower panels of Fig. 4 show the spatial extension of the anterior arcuate fasciculus, as represented in the BCB Toolkit, in relation to the *meta*-analytic clusters. It indicates the severity of the white matter tract disconnection effected by the lesions of the PTI subgroup. In summary, Fig. 4 shows that the lesions of the PTI subgroup impact severely the anterior arcuate fasciculus, implicating the anterior supramarginal gyrus and disrupting the connection to the ventral PMC and posterior BA 44. Extending from the posterior supramarginal gyrus to more anterior cortical fields than the anterior arcuate fasciculus, the SLF III is also affected by the white matter disconnections in the PTI subgroup. Hardly discriminable at the macroscopic level, the interlacing of this tract with the anterior arcuate fasciculus is addressed in Figure S3 of Supplementary Material. Computed as fractional overlaps of lesion with white matter tracts with the Tractotron function of the BBC Toolkit, a quantitative comparison of the disconnections between the PTI and RTI subgroups is shown in Table 4. The comparison shows that the essential distinction between the two subgroups stems from the differing overlaps of the anterior arcuate fasciculus and superior longitudinal fasciculus (SLF III). The overlaps with the corpus callosum are quantitatively minor (Table 4), but interfering with a site critical for haptic information transfer between hemispheres, ie at the border of the posterior corpus callosum and splenium (Fabri and Polonara, 2013).The functional significance is substantiated by the matching task data presented in Table 4. For individual overlaps, see Supplementary Material.

**Table 4:** Proportional overlap of lesions with white matter tracts. Listed are the fractional overlap of the lesions of the persistently impaired (PTI) and the recovered (RTI) groups with the arcuate and superior longitudinal fasciculi and corpus callosum. P* denotes the probability of lesion voxel values within a tract. The proportions for each tract were submitted to a one-way ANOVA and to 2-tailed t-tests, denoted by **. After Bonferroni correction for multiple comparisons, the significant t-tests retained significance at level, p < 0.05.

Subgroup	PTI (n = 7)		RTI (n = 7)			TN	RTI vs PTI		RTI vs TN		PTI vs TN	
*Tract*	P*	Proportionof lesion mean (SD)	P*	Proportionof lesion mean (SD)	P*	Proportionof lesion mean (SD)	p**		p**		p**	
							2-tailed *t*-test		2-tailed *t*-test		2-tailed *t*-test	
							t	p≤	t	p≤	t	p≤
Anterior Arcuate Fasc.	1	0.68 (0.21)	0.95	0.27 (0.24)	0.72	0.1 (0.15)	3.4	0.01**	2.1	0.04	7.9	0.0001**
SLF III	1	0.52 (0.22)	0.94	0.21 (0.19)	0.84	0.09 (0.13)	2.8	0.02	1.2	0.06	6.4	0.0001**
Corpus Callosum	1	0.08 (0.05)	0.94	0.04 (0.02)	0.99	0.02 (0.02)	2.0	0.07	1.8	0.09	4.5	0.0001**
ANOVA						Anterior Arcuate Fasc		SLF III		Corpus Callosum		
						F	p<	F	p<	F	p<	
						24.6	0.0001	17.9	0.0001	9.52	0.001	

**Fig. 5:**
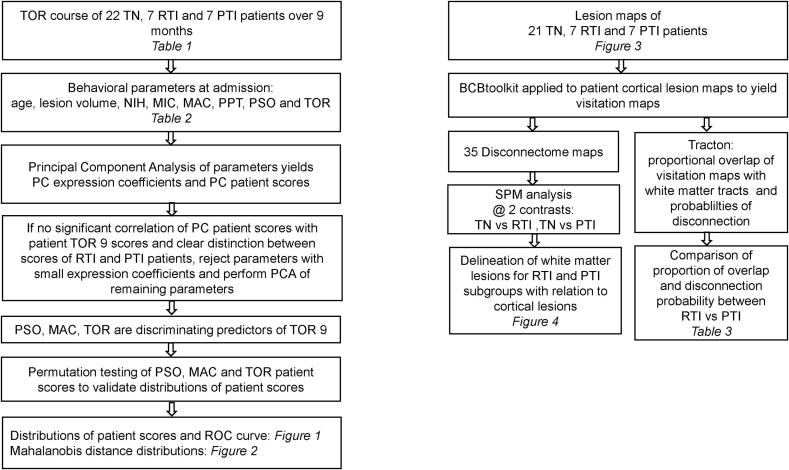
Depicted is a diagram of the analyses of behavioral data on the left and neuroimaging data on the right and the relationship to tables and figures, indicated by italics.

A graphical representation of the preceding assessments from patient subgroups to results is shown in Fig. 5.

### 3.3 Analysis of tactile matching task

The supplementary analysis of the matching task showed differential transfer of haptic information between affected and non-affected hands in RTI and PTI subgroups.

The matching tasks involving sequential explorations with the same hand, column 1 and 2 in Table 5, required information processing in only one cerebral hemisphere, whereas the explorations with alternative hands, column 3 and 4 in Table 5, required information processing in both hemispheres and haptic information transfer between them. The unilateral aH – aH exploration sequence served as reference for the alternated hand tasks in each subject at each visit in order to assess the contribution of interhemispheric information transfer to and from the affected hand. (1) Exploring with the same hand, the matching tasks involving the unaffected hand were unremarkable in both the PTI and RTI subgroups. The matching tasks involving the affected hand showed poor performance across the entire time course in the PTI patients, with neither explicit nor implicit identification of the replica, while significantly improving with time in RTI patients. (2) Exploring with sequentially alternated hands, the matching task showed improved performance at each assessment in the RTI patients while using the unaffected hand after presentation of objects to the affected hand, ameliorating over time from admission to the end of observation at 9 months; in the reverse direction haptic information was functional from admission. Thus, the haptic information transfer was effective in both directions of interhemispheric pathway at the end of the study. In the PTI subgroup haptic information was significantly ameliorating and effective only unidirectional across the interhemispheric path from the unaffected to the affected hand at the end of observation at month 9, whereas the implicated cortex was still unable to provide a recognizable haptic information for interhemispheric transfer. This increasing improvement in TOR performance, mediated by information transfer from the contralateral, non-affected hand, suggests functional recovery with time of the crossing interhemispheric pathway while haptic information processing in the affected IPL remained poor.

**Table 5:** Tactile matching tasks involving affected (aH) and unaffected (uH) hands. Tactile matching tasks were separated in (1) matching by two sequential explorations with the same hand (i.e. affected hand, aH, or unaffected hand, uH) and (2) matching by two sequential explorations with alternated hands (aH followed by uH or uH followed by aH). Each specific test consisted of the identification of 10 objects by finding the replica out of 5 alternative objects while the subjects were blinded. The results are displayed for admission and after 3 and 9 months. The first entry is the median of correct identification for the group; in parentheses is the range. Statistical analyses of all sequences were performed with the Friedmann test and comparisons of two sequences with the Mann-Whitney 2-tailed test. In these comparisons only p-values surviving correction for multiple comparisons according to Bonferroni were listed.

	**sequence**	1 uH: uH	2 aH: aH	1 vs 2	3 aH: uH	3 vs 2	4 uH: aH	4 vs 2	All: 1 2 3 4
**group**	**visit**			M−W		M−W		M−W	Friedmann
PTI n = 7	admission	8 (5–10)	0 (0–0)	z = 3.1 p < 0.005	3 (0–4)	n.s.	5 (1–8)	z = 3.1 p < 0.005	χ^2^ = 16.0 p < 0.002
	3	10 (5–10)	0 (0–5)	z = 3.0 p < 0.005	4 (0–6)	n.s.	5 (5–10)	z = 2.8 p < 0.005	χ^2^ = 16.6 p < 0.001
	9	10 (9–10)	6 (0–8)	z = 3.1 p < 0.005	5 (0–9)	n.s.	9 (7–10)	z = 2.8 p < 0.005	χ^2^ = 15.0 p < 0.002
Friedmann	over 9 months	n.s.	n.s.		n.s		χ^2^ = 10.5 p < 0.01		
RTI n = 7	admission	9 (4–10)	1 (0–8)	z = 2.56 p < 0.02	3 (0–8)	n.s.	8 (5–10)	z = 2.5 p < 0.05	χ^2^ = 12.0 p < 0.01
	3	9 (5–10)	6 (0–10)	n.s.	6 (0–9)	n.s.	9 (5–10)	n.s.	χ^2^ = 9.7 p < 0.05
	9	10 (5–10)	8 (7–10)	<0.02	9 (4–10)	n.s.	10 (9–10)	n.s.	χ^2^ = 8.01 0 < 0.05
Friedmann	over 9 months	n.s.	χ^2^ = 11.2 p < 0.005		χ^2^ = 11.2 p < 0.005		n.s.		

## 4 Discussion

The primary aim of this study was to investigate indicators to predict the course of recovery from tactile agnosia after first ischemic sensori-motor stroke in two subgroups of seven patients each out of a cohort of 36 post-stroke patients: those recovering from impairment (RTI) and those persistently impaired (PTI) after nine months.

The behavioral measure of tactile object recognition, TOR, is related to a natural manual skill of active touch in the macroscopic domain, acquired in early childhood (Jones & Lederman, 2006). The underlying motor control is present in everyday multifinger tasks involving specifically tactile object manipulation and object exploration (Abela et al., 2019, Weder et al., 1998). Their functionality is especially vulnerable to ischemic lesions and can lead to serious activity limitations in daily life (Miller et al., 2010, Veerbeek et al., 2011). This vulnerability is due to the complex sequential motor acts adapted to the explored objects, which requires temporally synchronous dynamic movements as well as concomitant asynchronous dynamic movements across distinct groups of finger joints. This motor sequence is termed finger gaiting and secures objects within the hand during exploration as has been verified using digital data gloves (Krammer et al., 2020). Roland and Mortensen detailed this type of human somatosensory exploration in the form of a model with input–output relationships during tactile exploration, encompassing kinesthesia, macrogeometry, size and shape (Roland & Mortensen, 1987).

The patients of the two groups exhibited apperceptive tactile agnosia at admission; they constituted subgroups of a cohort of 36 patients suffering first ischemic sensori-motor stroke. As shown in Table 1, the TOR performance scores measured at three and nine months showed no significant change in the unaffected hand, whereas the affected hands of the RTI and PTI subgroups show significant impairment at admission in both subgroups but after nine months only in PTI. In the study of this cohort using MVPA of the cortical lesion maps derived from DWI MRIs, we were able to distinguish the 22 TOR normal patients who did not suffer tactile agnosia from the PTI subgroup (Abela et al., 2019, Brodersen et al., 2010) regarding the recovery of tactile object recognition performance over nine months. However, the TOR recovery courses of the RTI subgroup overlapped those of both the TN and the PTI subgroup, preventing the use of the MVPA analysis to distinguish the RTI and PTI subgroups. Moreover, the TOR recovery courses that served as targets of the MVPA are derived from TOR performance during nine months, whereas only distinguishing RTI from PTI patients at admission could be clinically relevant. Thus, the purpose of the present study was to distinguish the TOR performance of two subgroups after nine months using only behavioral and neuroimaging data available at admission. As shown in Table 2, the behavioral measured included clinical data: age, lesion volume and NIH, as well as somatosensory behavioral scores: MIC_0, MAC_0, PPT_0, PSO_0 and TOR_0 determined at admission, where “_0” denotes score at admission. A hierarchy of PCAs yielded PC patient scores that correlated with TOR performance after nine months, TOR9. The set of measures yielding clear discrimination between RTI and PTI subgroups was composed of MAC_0, PSO_0 and TOR_0. The second component of the PCA explained 30% of the variance and yielded a significant correlation, p < 0.01, with TOR9. The discrimination was significant at the level, p < 0.002, and PC patient score threshold of −3 yielded perfect discrimination. A test set derived by permuting the three behavioral scores within the patient subgroups indicated the reliability of the results. As shown in Fig. 1, Fig. 2, comparisons of PCA patient scores and Mahalanobis distance distributions derived from the permuted behavioral scores showed good agreement. The receiver operating characteristic curve derived from the permutation testing yielded an 80% probability of correct identifications, an 8% probability for false identifications and a balanced accuracy of 90% for discrimination of the two subgroups. It yielded a second PC patient score threshold of −1.09.

The importance of PSO and MAC behavioral scores was indicated in our regression analysis of the same patient cohort relating TOR recovery courses to clinical and behavioral measures (Abela et al. 2019), in which these two scores were the most significant predictors. In that study, we also showed that the Mahalanobis distances derived from the 3-dimensional space of PSO9, MAC9 and TOR9 clearly distinguished among the TN, RTI and PTI subgroups. A Gaussian Mixture model confirmed the discrimination. For details, see Supplementary Material. In the current study, we show in Fig. 2 the considerable overlap of analogous Mahalanobis distances determined at admission for RTI and PTI subgroups, an additional indication of the reliability of the permutation testing. Behaviorally, PSO reflects the capability of adapting grasp configurations to familiar objects and MAC the ability to discriminate object size differences (Bohlhalter et al., 2002, Hömke et al., 2009). Together they reflect interdependence of exploratory action and perception (Dijkerman & de Haan, 2007). Notably, PSO, associated with the lateralized fine motor skill, exhibited the greatest expression coefficient in the first PC, whereas TOR, associated with shape and texture recognition showed the largest expression coefficient in the second PC. Concerning TOR_0, the importance of an initial test score in predicting the development of the test score over time has been observed also in other studies, e.g. in recovering the ability to swallow (Galovic et al., 2019). The applied exploration mode of spatial multifinger exploration, specific for macroscopic spatial object recognition, has been shown to be disrupted predominantly by the lesion of the supramarginal gyrus in the PTI subjects (Abela et al., 2019), whereas recognition of microscopic texture features, dependent on scanning movements (Morley et al., 1983), was less effected.

The impairment of MIC and other material features is most likely due to the combined SI and SII (OP1) lesions (Kitada et al., 2019; Sathian et al., 2011; Stilla & Sathian, 2008). However, despite the evidence based on the cortical lesion pattern, the pattern consisting of MIC, PSO and TOR did discriminate between the PTI and RTI subgroups, but at a lower level of significance than the pattern of MAC, PSO and TOR.

The clinical relevance of this procedure for analyzing behavioral data lies in the possible predictive use of the component expression coefficients. Just as projection of the PC expression coefficients onto the permuted behavioral measures yielded simulated patient scores, so could the measures of patients assessed at admission be used to predict those who would probably remain permanently impaired. Moreover, as more TOR assessments after nine months become available, the component expression coefficients could be refined.

With regard to the neuroimaging data, our previous study of the same patient cohort evidenced in the lesion maps of the PTI subgroup complete segregation at the cortical level of the anterior parietal lobule, mainly subarea PFt, and parietal operculum, subarea OP1 (Abela et al., 2019). The patients presenting no tactile agnosia evidenced no such lesions. Motivated by the goal of distinguishing the RTI and PTI subgroups in order to improve predictability of TOR recovery after nine months, we investigated disruptions of the white matter tracts impacted by the primary lesion area. The analysis should lead to a superior assignment of the related lesion pattern (Kessner et al. 2021). A secondary aim was the identification of the interhemispheric white matter pathway between the crucial lesion in the parietal cortex and the homotopic cortical area in the contralateral hemisphere, which might play a role in the recovery of TOR function.

Using the Tractotron and Disconnectome maps functions of the Brain Connectivity and Behavior Toolkit (Foulon et al., 2018), we found severe white matter disintegration in the anterior arcuate fasciculus in the PTI subgroup, but only slight disintegration in the same tract of the the RTI subgroup as shown in Table 4. The compact and contiguous SLF III, seems to be also affected, but is hardly discernible at the site of the fronto-parietal operculum by patho-anatomical means (Martino et al., 2011). In fact, there is a recent debate about equivalence of the SLF III to the arcuate fasciculus in its anterior or horizontal segment (Nakajima et al., 2020). This white matter disintegration is a new finding, shown in Table 4 and Fig. 4, concerning the tactile object recognition tasks, and confirms the need for differential evaluation of both grey and white matter possibly underlying somatosensory deficits after ischemic stroke.

According to both the common lesional network and its pattern in the PTI subgroup, the focus of the lesion lies in the mirror network (Caspers et al., 2006). A severely lesioned node of the cortical network delineated in a *meta*-analysis of 45 studies (Abela et al., 2019), the rostral IPL is reciprocally connected to Broca’s area (BA 44) and to the neighbouring vPMC in humans and macaque monkey homologue areas (Catani et al., 2005; Gregoriou et al., 2006). However, Kelly et al. (2010) found evidence for distinct resting state functional connectivity (RSCF) between the rostral supramarginal gyrus and ventral area 6, the latter distinct from that in Brodmann area 44. This may be due to differential connections from compartments of Brodmann area 44 to the posterior IPL, the latter providing semantic information with respect to control of motor actions (Frey et al., 2008), and from vPMC to rostral IPL. We posit the anterior arcuate fasciculus and associated SLF III as the most likely structural pathway underlying the related differential information transfer. Clos et al. (Clos et al., 2013) have characterized compartments in BA 44 involved in non-language related functions, e.g. in execution of action and perception as part of the mirror-neuron system and in higher cognitive functions. In fact, the posterior inferior sub-area in the frontal gyrus (pars opercularis), a conjectured target zone as well as exhibiting a diversity of motor functions, is also a part of the *meta*-analytic neuronal network elaborated above. In our previous study, we supposed that dysfunctional reciprocal linkage between anterior IPL and frontal motor areas disturbs combined processing of actions, like grasping, and perception underlying tactile object recognition (Fogassi and Luppino, 2005, Rizzolatti and Fogassi, 2014). Specific motor functions of posterior inferior Broca area encompass learning of motor finger sequences (Seitz & Roland, 1992); visual motor learning (Toni et al., 2001); precision grip (Ehrsson et al., 2001); matching of hand posture configurations in accordance with visual and functional demands (Vingerhoets et al., 2013); and observation and imitation of actions as well as imagination or observation of motions (Binkofski & Buccino, 2004). The role of the lesioned OP1 may be related do a decay in complex information processing during tactile working memory as part of the mirror neuron system as well as in processing of rather microscopical texture features (Caspers et al., 2010).

We suggest that, combined with the lesions in the anterior IPL and OP1, axonal damage to anterior arcuate fasciculus in PTI subjects interrupted completely transfer of haptic information from the anterior IPL to premotor cortices in the inferior frontal gyrus and possibly recursive feedback tracts from them. In contrast, the isolated tract lesions of arcuate fasciculus in RTI subjects were not severe enough to prevent recovery of function in the affected hand, as indicated by the significantly improving TOR performance shown in Table 1.

Insight into the inter-hemispheric transfer of haptic information was also provided by the supplementary analysis of the matching task. The matching task with the affected hand showed in the PTI subgroup severe impairment over the entire nine months, whereas the RTI subgroup showed significant improvement. The matching task with sequentially alternated hands showed additional discrimination. The PTI group showed improvement only in matching from the non-affected hand to the affected hand, whereas the RTI group regained normal bidirectional function of haptic information transfer. The unidirectional haptic information transfer from the unaffected to the affected hand in the PTI subgroup indicates functional restoration of the lesioned inter-hemispheric path at the border of the posterior corpus callosum and splenium (Fabri & Polonara, 2013). On the contrary, the severely dysfunctional and segregated anterior IPL remained incapable of providing a discernible haptic information to the homotopic area of the contralateral hemisphere. This observation may have a potential impact on rehabilitation strategies.

Limitations are the rather small sample size of both the recovered and persistently impaired TOR subgroups. Despite the small sample size, a typical issue for rare disorders like tactile agnosia, the PCA identified a pattern of sensitive sensori-motoric measures. Moreover, the results of the PCA were validated by a test set based on permutation of patient behavioral scores, leading us to expect that the model generalises to larger patient cohorts. This procedure thus represents an approach to cope with the small sample size. Another possible limitation is the indirect determination of the affected tracts based on 10 healthy individuals of a different age range. However, previous studies have established this method, and submitting patients at admission to the lengthy DWI acquisitions required for direct determination would be a serious imposition. The findings reported here are valid for apperceptive tactile agnosia due to an elementary sensori-motor disorder, but not for associative tactile agnosia, which may dependent rather on a lateralized neuronal network to the right hemisphere (Balsamo et al., 2008, Platz, 1996, Veronelli et al., 2014). The elementary multifinger task underlying extraction of somatosensory information is independent of fast and dynamic exploration and of brain lateralization (Krammer et al., 2020, Craddock and Lawson, 2009, Yamashita, 2015). In contrast, tool use involves similar anterior parietal areas, but predominantly in the left hemisphere (Orban and Caruana, 2014).

## 5 Conclusion

Our study has found two patterns that can be used to predict at admission if a patient can be expected to recover from apperceptive tactile agnosia after first ischemic stroke. The behavioral pattern consists of the PCA expression coefficients derived from three measures of daily tactile functionality: PSO_0. MAC_0 and TOR_0. A test set derived from permutation of behavioral scores indicated 90% accuracy of the discrimination between permanently impaired and recovering subgroups. The neuroimaging pattern relies on the combined analysis of both cortical lesions and implicated white matter tracts. The distinguishing feature is the overlap of the lesions with the anterior arcuate and associated the superior longitudinal fasciculus. Our evaluation implied a probability for correct discrimination between RTI and PTI subgroups of better than 98%. The combined analysis improved the balanced accuracy of distinguishing NT from RTI and PTI subgroups from 86% in our MVPA analysis to essentially 100%. The supplementary analysis evaluated the possible role of haptic information transfer between hemispheres in supporting the recovery from tactile agnosia.

## Declaration of Competing Interest

The authors declare that they have no known competing financial interests or personal relationships that could have appeared to influence the work reported in this paper.

## Data Availability

Data will be made available on request.
