# Supplementary material for: Distinguishing transient from persistent tactile agnosia after partial anterior circulation infarcts – Behavioral and neuroimaging evidence for white matter disconnection
Source: Neuroimage Clin. 2022 Sep 13;36:103193. doi: 10.1016/j.nicl.2022.103193 (PMC9486662; doi:10.1016/j.nicl.2022.103193)

Distinguishing transient from persistent tactile agnosia after partial anterior circulation infarcts - Behavioral and neuroimaging evidence for white matter disconnection

John H. Missimer, Eugenio Abela, Manuela Pastore-Wapp, Roland Wiest, Bruno J. Weder

Supplementary Material

- Tactile Object Recognition task
- Principal component analysis of behavioral data
- Mahalanobis distances and Gaussian cluster model
- Proportional overlaps of white matter tracts with lesions
- Tractotron results concerning white matter tract lesions using BCBtoolkit
- The interrelation between anterior arcuate fasciculus and SLF III – a classification issue

Tactile Object Recognition task

Table S1: Objects used for tactile object recognition task

Marble	Clothes peg	Nutshell	Tape reel	Thimble	Eraser
Pencil	Button	Coin	Key	Battery	Ring
Sharpener	Glue stick	Tippex	Wooden plug	Bottle opener	Screw
Paperclip	Velcro	Pebble	Rubber band	Dice	Nail
Hook	Ball pen	Block battery	Shim	Syringe (5 ml)	Key ring

Principal component analysis of behavioral data

Table S2. Patient data used in principal component analysis expressed as z-scores

patient	age	lesvol	NIH 0	PSO 0	MAC 0	MIC 0	PPT 0	TOR 0	TOR 9
RTI1	0.6200	0.4653	6	13.48	-14.3	-22.30	-18.60	-57.20	-15.20
RTI2	1.0396	1.1997	4	6.77	-21.00	-19.40	-113.40	-59.20	-11.20
RTI3	1.2074	1.9091	5	4.17	-39.30	-33.70	-48.10	-59.20	-9.20
RTI4	-1.2261	-0.1321	4	58.82	-6.00	-2.30	-0.70	-37.20	-21.20
RTI5	0.7879	0.0173	3	14.40	-22.70	-33.70	-58.30	-35.20	0.80
RTI6	0.7879	2.0585	4	27.60	-16.00	-10.90	-34.20	-51.20	-9.20
RTI7	1.3752	-0.4744	3	9.12	-9.30	-5.10	-8.40	-19.20	-9.20
PTI1	-1.0582	2.0585	6	20.1	-19.30	-15.10	-26.30	-59.20	-59.20
PTI2	-0.8065	16.2601	14	51.28	-14.30	-33.70	-100.20	-59.20	-59.20
PTI3	-0.9743	0.9072	6	27.56	-39.30	-33.70	-112.40	-59.20	-39.20
PTI4	0.3683	2.9298	6	5.85	-39.30	-33.70	-112.40	-59.20	-39.20
PTI5	-1.3939	4.5105	3	5.35	-39.30	-33.70	-48.10	-59.20	-39.20
PTI6	0.1165	0.6832	11	38.87	-14.30	-33.70	-100.20	-53.20	-55.20
PTI7	-1.0582	1.3864	11	22.70	-39.30	-33.70	-19.60	-59.20	-53.20

Table S3. Relevant features of PCA comprising PSO 0, MAC 0 and TOR 0. Corr coef are the correlation coefficients of TOR 9 with the patient scores, and p the corresponding probabilities.

	% explained	Corr coef	p
PC1	62	-0.12	0.69
PC2	30	0.67	0.008

Table S4. Patient scores for PC2 of PCA comprising PSO 0, MAC 0 and TOR 0. These are plotted in Figure 1.

recovered	PC1	PC2	permanent	PC1	PC2
RTI1	-3.23	3.79	PTI1	-1.02	-3.01
RTI2	-12.69	3.06	PTI2	26.82	-16.83
RTI3	-24.35	-2.64	PTI3	-5.42	-14.44
RTI4	43.18	-0.43	PTI4	-22.99	-3.48
RTI5	-0.95	17.13	PTI5	-23.40	-3.23
RTI6	8.93	0.66	PTI6	18.40	-5.92
RTI7	6.09	37.31	PTI7	-9.36	-11.98

Table S5. The component expression coefficients describing the PC patterns

	PSO 0	MAC 0	TOR 0
PC1	0.8093	0.5221	0.2693
PC2	-0.5046	0.3831	0.7737

Table S6. Kruskal-Wallis ANOVA of PC2: RTI vs PTI

Source	SS	df	MS	$\chi 2$	$p > \chi 2$
Columns	171.5	1	171.5	9.8	0.0017
Error	56	12	4.667		
total	227.5	13			

Figure S1. Scatter plot of PC patient scores: PC2 vs PC1 for PTI (red) and RTI (blue).

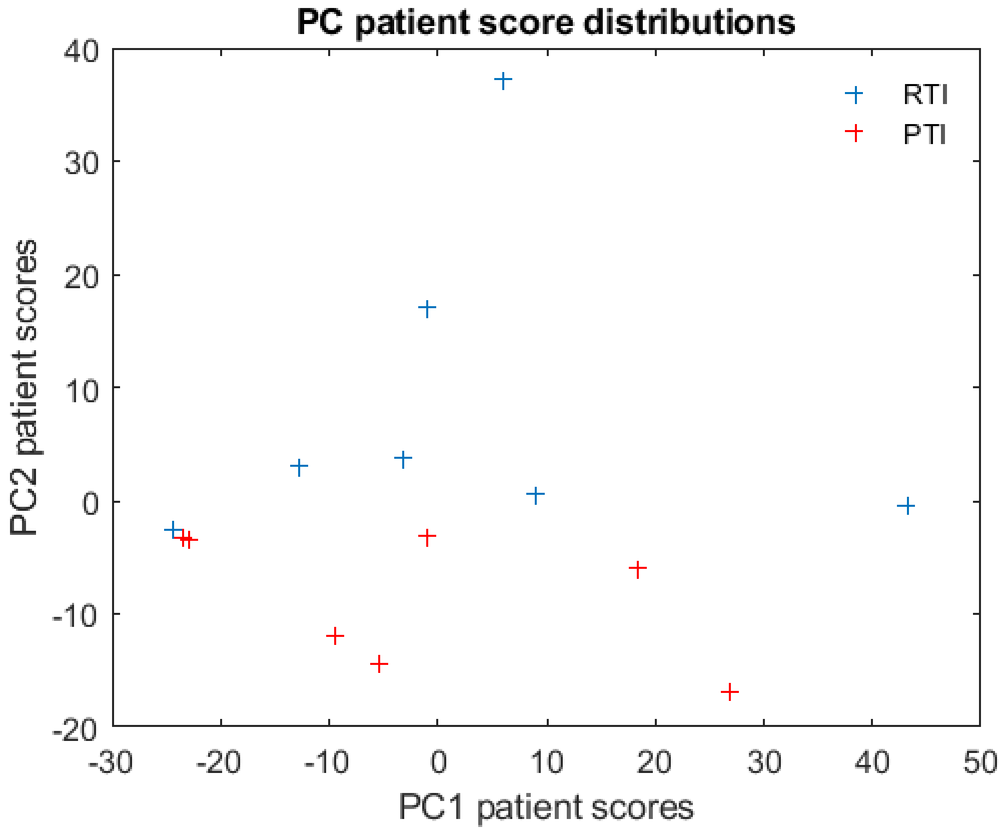

Mahalanobis distances and Gaussian mixing model

Table S6. Mahalanobis distances derived from PSO 9, MAC 9 and TOR 9 with respect to unimpaired patients at nine months and Gaussian mixture modeling

recovered	dist	permanent	dist
RTI1	179	PTI1	3004
RTI2	183	PTI2	3004
RTI3	542	PTI3	1862
RTI4	355	PTI4	1919
RTI5	16	PTI5	2038
RTI6	147	PTI6	3530
RTI7	93	PTI7	2382

Table S7. Gaussian mixing model derived from Mahalanobis distances of Table S5. With 21 iterations, the distribution with 3 component in 1 dimension yielded a log-likelihood of -192.3

Component	Mixing Proportion	Mean	Sigma
1 TN	0.544	1.551	1.94
2 RTI	0.261	163.220	165.87
3 PTI	0.195	2592.2	685.42

Figure S2. Histogram of Mahalanobis distances with Gaussians derived from the mixing model. The Gaussians have been rescaled to accommodate the histograms, but the relative heights of the Gaussians are correct. Green denotes TN; Blue denotes RTI; red PTI.

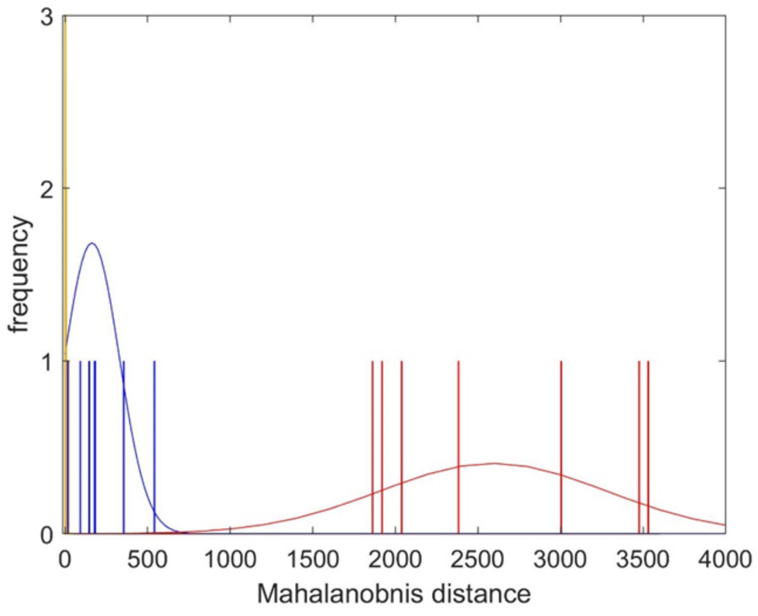

Proportional overlaps of white matter tract segments with lesions

Table S8. Proportions of overlap of lesion with white matter tract for patient subgroups used in Table 3 of manuscript.

	Anterior Arcuate Fasciculus AH	Superior Longitudinal Fasciculus III AH	Corpus_callosum
RTI1	0.597	0.395	0.056
RTI2	0.415	0.341	0.038
RTI3	0.562	0.487	0.075
RTI4	0.046	0.042	0.029
RTI5	0.159	0.125	0.007
RTI6	0.101	0.091	0.038
RTI7	0.005	0.002	0.009
PTI1	0.529	0.433	0.071
PTI2	0.993	0.942	0.169
PTI3	0.726	0.309	0.033
PTI4	0.770	0.514	0.052
PTI5	0.446	0.437	0.074
PTI6	0.846	0.669	0.116
PTI7	0.434	0.341	0.030

Table S9. Tractotron results (Foulon et al. 2018; http://www.toolkit.bcblab.com) showing probability (P) and mean proportion of overlap (OL) of lesion projection into white matter tracts of PTI, RTI and TN subgroups. The threshold P>0.5 indicates disconnection of a specific tract due to the lesion. AH denotes affected hand.

**PTI subgroup**	**Mean_P**	**Mean_OL**	**SD_OL**	**RTI subgroup**	**Mean_P**	**Mean_OL**	**SD_OL**
Anterior Arcuate Fasciculus AH	1.00	0.68	0.21	Superior_Longitudinal_Fasciculus_II_AH	1.00	0.20	0.11
Corpus_callosum	1.00	0.08	0.05	Cortico_Spinal_AH	0.99	0.14	0.07
Superior_Longitudinal_Fasciculus_III_AH	1.00	0.52	0.22	Fronto_Striatal_AH	0.96	0.06	0.03
Superior_Longitudinal_Fasciculus_II_AH	1.00	0.38	0.21	Handinf_U_tract_AH	0.96	0.45	0.26
Fronto_Insular_tract5_AH	0.99	0.78	0.29	Anterior Arcuate Fasciculus_AH	0.95	0.27	0.25
Handinf_U_tract_AH	0.97	0.72	0.29	Pons_AH	0.95	0.10	0.05
Arcuate_Long_Segment_AH	0.96	0.51	0.32	Corpus_callosum	0.94	0.04	0.02
Arcuate_Posterior_Segment_AH	0.95	0.51	0.39	Superior_Longitudinal_Fasciculus_III_AH	0.94	0.21	0.19
Cortico_Spinal_AH	0.92	0.12	0.11	Arcuate_Long_Segment_AH	0.92	0.20	0.15
Fronto_Striatal_AH	0.88	0.07	0.08	Frontal_Commissural	0.88	0.02	0.02
Fronto_Insular_tract4_AH	0.90	0.63	0.36	Anterior_Thalamic_Projections_AH	0.85	0.03	0.02
Superior_Longitudinal_Fasciculus_I_AH	0.88	0.06	0.06	Superior_Longitudinal_Fasciculus_I_AH	0.85	0.08	0.07
Frontal_Aslant_tract_AH	0.88	0.20	0.20	Handsup_U_tract_AH	0.80	0.24	0.21
Frontal_Commissural	0.87	0.03	0.03	Frontal_Aslant_tract_AH	0.80	0.04	0.03
Inferior_Fronto_Occipital_fasciculus_AH	0.86	0.07	0.13	Frontal_Superior_Longitudinal_AH	0.75	0.09	0.10
Handsup_U_tract_AH	0.85	0.34	0.21	Fronto_Insular_tract5_AH	0.75	0.47	0.46
Pons_AH	0.81	0.07	0.08	Handmid_U_tract_AH	0.64	0.37	0.36
Face_U_tract_AH	0.82	0.84	0.27	Cingulum	0.62	0.02	0.03
Frontal_Superior_Longitudinal_AH	0.81	0.06	0.06	Fronto_Insular_tract4_AH	0.59	0.28	0.34
Inferior_Longitudinal_AH	0.83	0.09	0.11	Face_U_tract_AH	0.53	0.32	0.37
Handmid_U_tract_AH	0.77	0.38	0.37	Frontal_Inferior_Longitudinal_AH	0.53	0.02	0.03
Frontal_Inferior_Longitudinal_AH	0.75	0.34	0.41	Arcuate_Posterior_ Fasciculus _AH	0.50	0.08	0.12
Anterior_Thalamic_Projections_AH	0.74	0.04	0.06				
Fronto_Insular_tract3_AH	0.74	0.40	0.41				
Optic_Radiations_AH	0.62	0.05	0.08				

NT subgroup	Mean_P	Mean_OL	SD_OL
Superior_Longitudinal_Fasciculus_II_AH	0.99	0.11	0.07
Corpus_callosum	0.99	0.02	0.02
Pons_AH	0.93	0.07	0.06
Fronto_Striatal_AH	0.93	0.04	0.04
Cortico_Spinal_AH	0.90	0.08	0.08
Superior_Longitudinal_Fasciculus_I_AH	0.90	0.04	0.06
Handsup_U_tract_AH	0.89	0.21	0.17
Frontal_Commissural	0.89	0.03	0.03
Anterior_Thalamic_Projections_AH	0.87	0.02	0.03
Handinf_U_tract_AH	0.86	0.19	0.21
Superior_Longitudinal_Fasciculus_III_AH	0.84	0.09	0.13
Frontal_Superior_Longitudinal_AH	0.81	0.07	0.12
Anterior Arcuate Fasciculus_AH	0.72	0.10	0.15
Frontal_Aslant_tract_AH	0.70	0.04	0.07
Arcuate_Long_Segment_AH	0.70	0.11	0.21
Handmid_U_tract_AH	0.62	0.14	0.20

The interrelation between anterior arcuate fasciculus and SLF III – a classification issue

Figure S3. Interweaving of ROIs, representing horizontal segments of the anterior arcuate fascicle and SLF III, illustrates the difficulty of differentiating between them at the macroscopic level. The white area in figure represents the anterior arcuate fasciculus, the red border marks SLF III and pale yellow to yellow varying degrees of intersection. The green areas show subareas of angular gyrus, parakeet subarea PGa and green pear subarea PGp. It becomes evident that SLF III extends more anteriorly and posteriorly to the posterior supramarginal gyrus and angular gyrus.

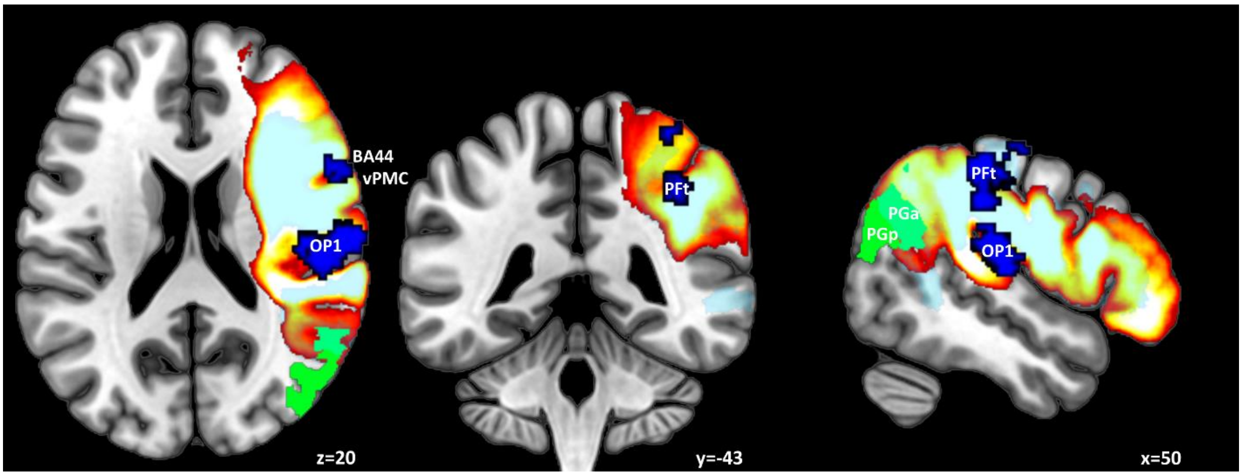

Supplement: Supplementary data 1 [file mmc1.pdf]
